# Spiking Neuron Mathematical Models: A Compact Overview

**DOI:** 10.3390/bioengineering10020174

**Published:** 2023-01-29

**Authors:** Luigi Fortuna, Arturo Buscarino

**Affiliations:** 1 Dipartimento di Ingegneria Elettrica Elettronica e Informatica, University of Catania, 95125 Catania, Italy; 2 IASI, Consiglio Nazionale delle Ricerche (CNR), 00185 Roma, Italy

**Keywords:** spiking neuron, nonlinear dynamics, neural network

## Abstract

The features of the main models of spiking neurons are discussed in this review. We focus on the dynamical behaviors of five paradigmatic spiking neuron models and present recent literature studies on the topic, classifying the contributions based on the most-studied items. The aim of this review is to provide the reader with fundamental details related to spiking neurons from a dynamical systems point-of-view.

## 1. Introduction

Spiking is a fundamental feature of neuronal dynamics, intrinsically ensuring efficiency and low-power consumption. The control and communication processes in natural environments are often driven by spiking signals. Spiking signals can be generated by elementary physical phenomena and, therefore, mathematical models and simple logic rules can be adapted to efficiently mimic their characteristics. For these reasons, spiking systems are receiving interest from the scientific community. This review paper summarizes the fundamental properties of commonly adopted neuron models and their dynamical characteristics; we present a compact overview of the recent literature results, organized according to the model.

**Figure 1:**
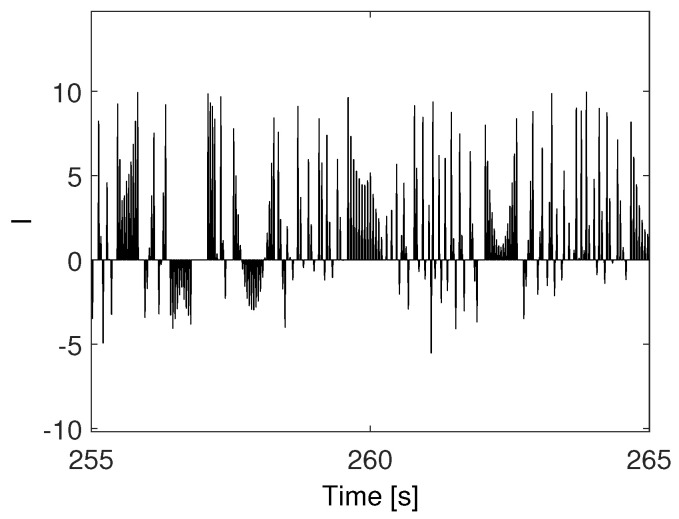
A spiking signal.

Spiking signal features essentially reflect the mechanisms of accumulation and fast energy release, characterized by slow–fast dynamics. An example of a spiking signal is presented in Figure 1. The mathematical model that generated such a spiking signal is given by the following set of nonlinear differential equations:

(1) $$\left\{\begin{array}{c} \dot{X}=Y\\ \dot{Y}=\frac{1}{J}\left(ISB\sin\left(X\right)-KY\right) \end{array}\right.$$

**Figure 2:**
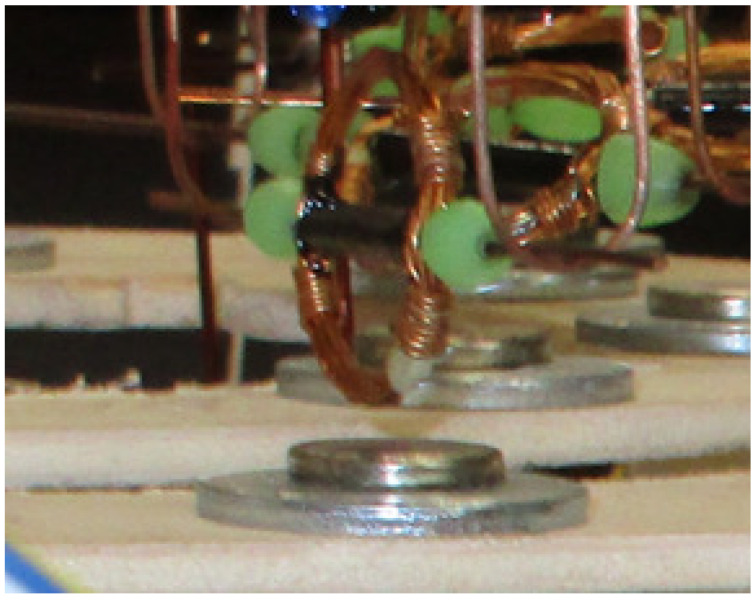
Coil rotating around its main axis, located on a powered trail above a small magnet.

This model represents the dynamics of a coil rotating around its axis, thanks to the interplay between a magnetic field and the current flowing through it [1], as in the experimental setup shown in Figure 2.

Referring to Equation (1), *X* represents the phase of the coil, *Y* the angular speed, *J* the angular momentum, and *K* the friction factor. *I* is the current flowing into the coil and is given by

$$I=\frac{V_{a}-YSB\sin\left(X\right)}{R(X)}$$

with $V_{a}$ being the voltage supplied to the coil, *S* the coil area, *B* the magnetic field, and R(X) the contact resistance, which, due to the coil construction constraint, is nonlinear according to:

$$R\left(X\right)=\left\{\begin{array}{cc} 0.2\Omega & \mathrm{if}X<\pi \\ 10k\Omega & \mathrm{if}X\geq \pi \end{array}\right.$$

Concerning the dynamic friction factor, the following nonlinear characteristics can be considered

$$K=\left\{\begin{array}{cc} K_{H} & \mathrm{if}Y<2Hz\\ K_{L} & \mathrm{if}Y\geq 2Hz \end{array}\right.$$

**Figure 3:**
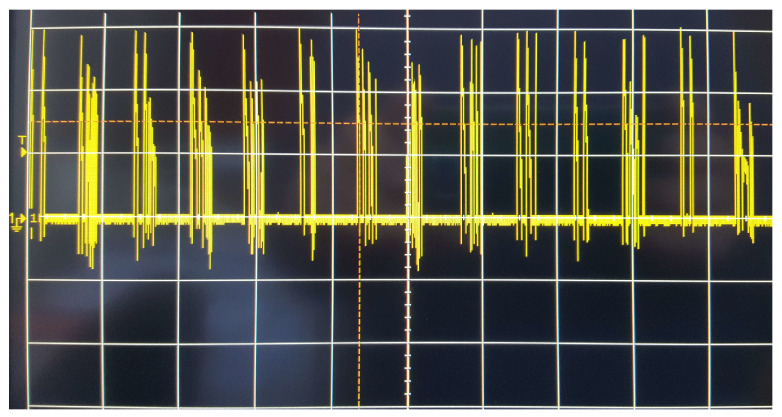
Spiking signal generated by the electromechanical dynamics of the rotating coil.

The experimental setup leads to the voltage trend reported in Figure 3, representing an example of the electromechanical spiking signal.

**Figure 4:**
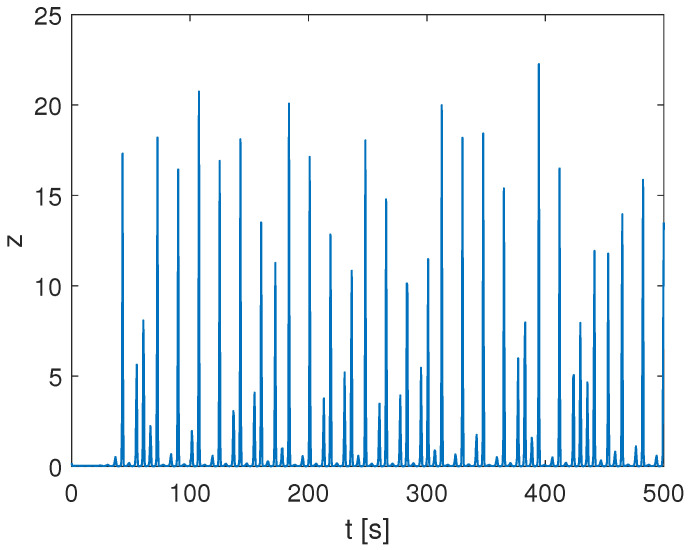
Spiking behavior in the chaotic Rössler oscillator.

Another example of a spiking signal can be obtained by considering the Rössler oscillator [2], described by the following set of equations

(2) $$\begin{array}{c} \dot{x}=-y-z\\ \dot{y}=x+ay\\ \dot{z}=b+z\left(x-c\right) \end{array}$$

with a=0.2, b=0.2, and c=5.7, leading to irregular spiking behavior for the variable *z* reported in Figure 4.

**Figure 5:**
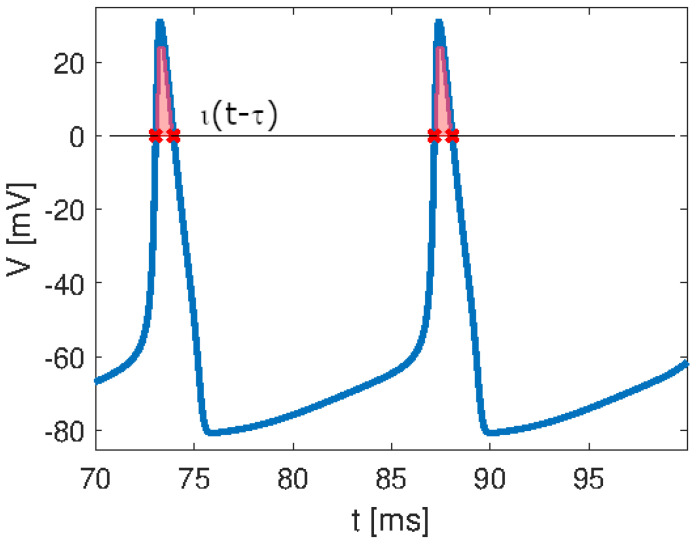
Spiking signal characterization: the function ι(t−τ) follows the spike when it overcomes the zero threshold. The shaded area represents the integral area of the spike that, analogous to the δ function, equals 1/a, with *a* being the sensitivity of the neuron.

Spiking signals are, therefore, ubiquitous as they are present in very different scenarios. They are the bases of neural information transfer. In order to provide a formal characterization of a spiking signal, an analogy with the Dirac impulse $\delta (t-t_{n})$ can be performed [3]. The Dirac impulse is defined as a pulse of infinite magnitude occurring at the instantaneous time $t_{n}$, thus having an integral area equal to 1. Analogously, a single spike, which in general is associated with the action potential of a biological neuron, can be defined through the function ι(t−τ) that assumes the value of the spike only when it overcomes a given threshold, as schematically reported in Figure 5. The integral area of the spike, i.e., the shaded region in Figure 5, is equal to 1/a, analogous to the δ function, where *a* is called the sensitivity of the neuron. Therefore, a spiking signal is analogous to an impulse train, with the latter consisting of pulses of infinitesimal duration and the former consisting of spikes of finite duration.

This contribution is motivated by the actual intense scientific activity related to spiking signals and spiking neural networks, and from the consequent intense activity in realizing spiking neuronal systems, with appropriate hardware and software tools.

In this paper, we present an overview of five paradigmatic mathematical models of neuron dynamics and their related bifurcation scenarios. The behavior of this class of neurons is a rich recipient of emerging dynamical behavior.

We present a complete overview of recent studies regarding the considered neuron models. In particular, papers related to the following main items involved in the study of neuron models are considered: parameter estimation, bifurcation and chaos, synchronization, stochasticity, noise, non-integer (fractional) order dynamics, implementations, and memristors.

In the conclusion, we present a discussion on the main hardware/software platforms involve spiking neurons, both for research studies and educational aims; we remark on the intense activities of spiking neurons that are evident from the studies reported on in the scientific journals.

## 2. Modeling Spiking Neurons and Their Dynamical Behaviors

### 2.1. Genesis of Neuron Modeling

Spiking neural networks are artificial networks that work similarly to natural neural networks. They are based on the concept that the network propagates information based on the principle of threshold levels. This means that each neuron activity is activated by other neurons, only if a given level for the membrane potential, called activation threshold, is passed. Under this condition, it is said that the neuron *fires*, generating further electrical activity that will contribute toward inducing other connected neurons to pass the activation threshold. The described mechanism is called *integrate and fire*.

Therefore, essential element spiking networks are proper choices in mathematical models for neuron dynamics, keeping in mind that, due to threshold behaviors, a neuron is the stereotype of a nonlinear dynamical system.

**Figure 6:**
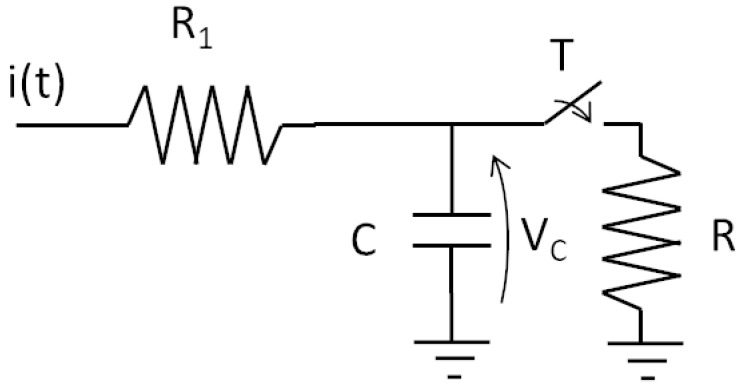
Equivalent circuit used by Lapique.

Researchers have focused on obtaining suitable models since the beginning of the last century. In fact, Lapique, in 1907 [4], developed a model of a neuron considering only the electrical behavior of an active membrane. The study paved the way for advanced models of spiking neurons. The Lapique model was conceived as an electrical circuit with one capacitor and two resistors, as shown in Figure 6.

The capacitor *C* plays the role of integration, while the resistor *R* ‘conducts’ when the switch is closed, i.e., the switch is controlled by the voltage $V_{c}$, thus allowing for the immediate discharging of the capacitor. This mechanism emulates the firing operation. Thus, we can characterize the resistor and the capacitor as membrane resistance and membrane capacitance.

This simple model is effective in emulating the neuron’s activation behavior. At the time of its publication, the Lapique model was successful in investigating the frequency response of a nerve coupled to a stimulating electrode. The simple and useful Lapique model has been widely used to represent an impressive example of a membrane activation mechanism. The concept of the Lapique model is still gaining interest, as witnessed in [5] and a recent translation (from French) of the original article by Lapique [6]. Thanks to Lapique’s work, the principle of the electrical excitation of nerves was formalized by Nernst in 1904 [7], establishing the bioelectric basis of excitable membranes.

**Figure 7:**
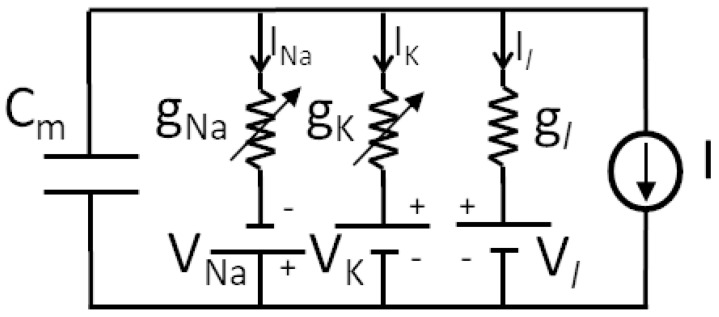
Equivalent circuit used by Hodgkin and Huxley.

Studies by Hodgkin and Huxley (HH) [8] allowed establishing the first biophysically complete dynamical model of a neuron, where membrane conductance was explicitly considered to generate action potentials. It was derived from experimental studies on giant nerve fibers and based on the nonlinear electrical scheme presented in Figure 7, providing an accurate mathematical model.

The FitzHugh–Nagumo model (FHN) is a simplified second-order model of the HH model. It was proposed in 1961 [9]. In its original form, it gives both the active behavior of the neuron and an oscillating aperiodic behavior when it is forced by an external periodic wave.

The Hindmarsh–Rose model (HR) was described as a further simplification of the HH model in 1984 [10] where the complex nonlinearities were approximated thanks to experimental observations that led to an optimal representation based on second- and third-order polynomial expressions. Today, the HR model is one of the key reference models; it is complete and reliable, especially when used in the implementation of neutron networks [11] and studying synchronization problems [12].

A very simple behavioral model of a neuron that allows emulating spiking and bursting (i.e., intense sequences of spiking) phenomena, for more classes of cortical neurons, was proposed by Izhikevich in [13]. The simplicity and parametrization of the model (based on a few quantities that allow for the descriptions of neuronal cortical behaviors in more conditions) make the Izhikevich model suitable for the implementation of neurocomputing platforms with high numbers of neurons. From a dynamical systems perspective, in [13], a gallery of peculiar spiking behaviors is presented.

Another appreciated mathematical model for spiking neurons is the Morris–Lecar model (ML) proposed in 1981 [14]. It gives a second-order approximation of the HH model. The model essentially describes the membrane voltage *V* and the recovery variable *N* that gives the probability that the potassium channel conducts. Both the simplicity and the biophysical meanings of the variables make this model appropriate for more relevant studies [15].

### 2.2. Mathematical Models and Bifurcations

We now outline the mathematical equations governing the dynamics of the previously discussed neuron model. To this aim, we summarize the main parameters and their physical meanings. All parameters are reported as dimensionless quantities. In order to uncover the role of the driving current, we focus on the bifurcation diagrams with respect to the current parameters. To this aim, the dynamics of spiking neurons are often characterized in terms of the interspike interval (ISI), i.e., the time span between two subsequent spikes. When a chaotic spiking arises, it is more convenient to observe the bifurcation route by plotting the local minima and maxima, a strategy adopted for chaotic nonlinear dynamical systems [16].

Let us consider the HH model; it is described by the following set of differential equations:

(3) $$\begin{array}{c} \dot{V}=\frac{I_{ion}}{C}\\ \dot{n}=\alpha_{n}\left(1-n\right)-\beta_{n}n\\ \dot{m}=\alpha_{m}\left(1-m\right)-\beta_{m}m\\ \dot{h}=\alpha_{h}\left(1-h\right)-\beta_{h}h \end{array}$$

where $I_{ion}=I-I_{K}-I_{Na}$, with $I_{K}=g_{K}n^{4}\left(V-E_{K}\right)$ and $I_{Na}=g_{Na}m^{3}h\left(V-E_{Na}\right)$, and

(4) $$\begin{array}{c} \alpha_{n}=0.01\frac{\left(10-V\right)}{e^{\frac{10-V}{10}}-1}\\ \beta_{n}=0.125e^{-\frac{V}{80}}\\ \alpha_{m}=0.1\frac{\left(25-V\right)}{e^{\frac{25-V}{10}}-1}\\ \beta_{m}=4e^{-\frac{V}{18}}\\ \alpha_{h}=0.07e^{-\frac{V}{20}}\\ \beta_{h}=\frac{1}{e^{\frac{30-V}{10}}+1} \end{array}$$

The model consists of four differential equations, the first consisting of the membrane’s potential dynamics and the other consisting of the dynamics of the probability of the potassium channel activation, the sodium channel activation, and the sodium channel inactivation, respectively. To this aim, the functions $\alpha_{i}$ and $\beta_{i}$ are defined according to the Boltzmann transport equations, as in (4). The current $I_{ion}$ contains the input current *I* and the currents related to the sodium and potassium channels.

**Figure 8:**
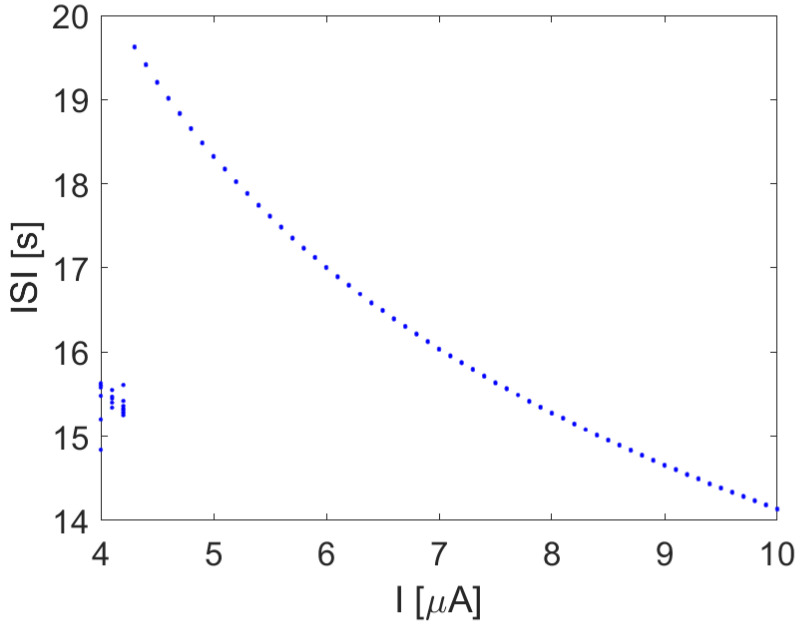
ISI in the Hodgkin–Huxley model with *I* constant.

When the input current *I* is a constant value, a transition toward a spiking behavior occurs, as reported in Figure 8.

**Figure 9:**
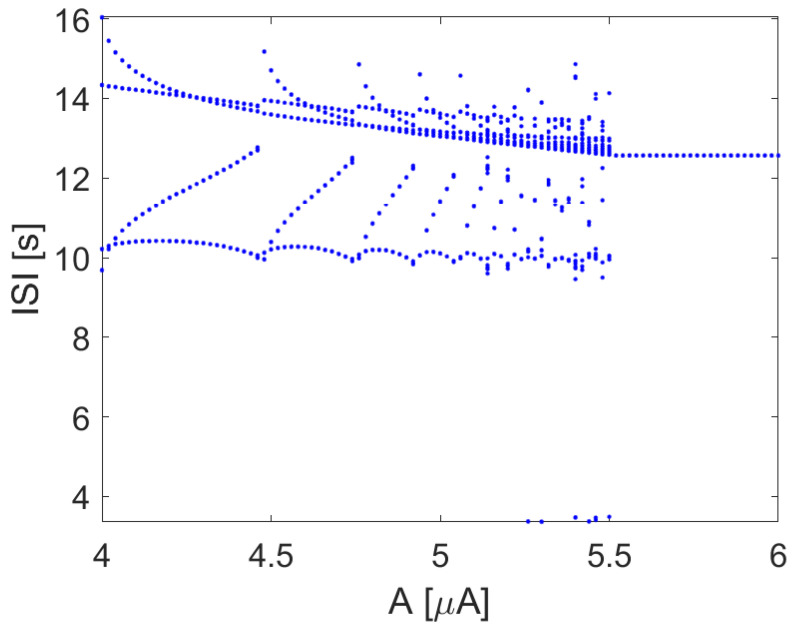
ISI in the Hodgkin–Huxley model with I=A(1+sinωt), with ω=0.5 rad/s. Other parameters: $E_{K}=-12$, $E_{Na}=115$, C=1, $g_{k}=36$, $g_{Na}=120$.

When the input current *I* is a sinusoidal signal with constant frequency, varying the amplitude *A* leads to the bifurcation diagram, as reported in Figure 9, showing the emergence of complex patterns of spiking, including windows of chaotic behavior.

**Figure 10:**
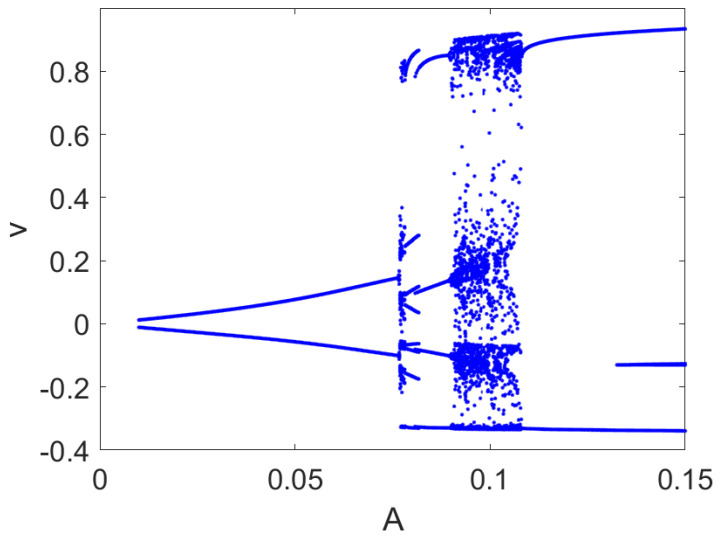
Bifurcation diagram of the FHN model. Local maxima and minima of the variable *v* are reported for each value of *A* with ω=0.1271 rad/s. Other parameters: a=0.1, b=1.

The FHN model is characterized by the following set of differential equations:

(5) $$\begin{array}{c} \dot{v}=v\left(v-1\right)\left(1-av\right)-w+I_{ext}\\ \dot{w}=bv \end{array}$$

where *v* is the membrane voltage, *w* is the recovery variable, and $I_{ext}=\frac{A}{\omega }\cos\omega t$ is the driving current. The effect of the current $I_{ext}$ is reported in the bifurcation diagram in Figure 10, where a wide range of chaotic spiking behaviors can be retrieved.

**Figure 11:**
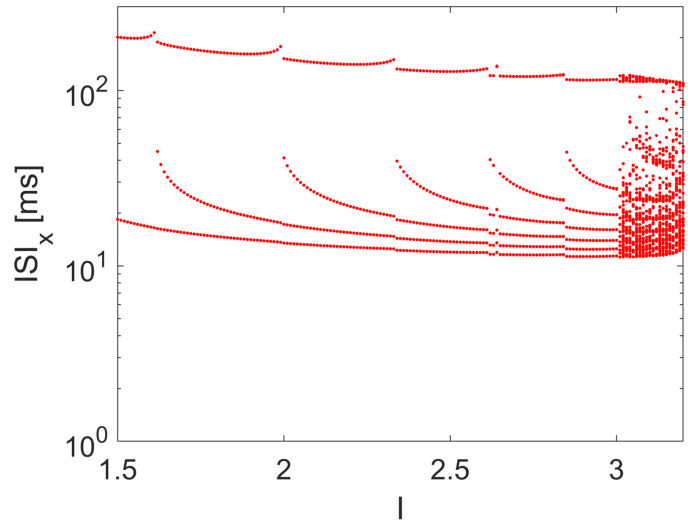
ISI bifurcation diagram of the HR model with respect to *I*. Other parameters: a=1, b=3, c=1, d=5, r=0.003, s=4, and $x_{R}=-\frac{8}{5}$.

The dynamics of the Hindmarsh–Rose (HR) model are reported as follows: [12]:

(6) $$\begin{array}{c} \dot{x}=I+y-z+bx^{2}-ax^{3}\\ \dot{y}=c-dx^{2}-y\\ \dot{z}=r\left(s\left(x+x_{R}\right)-z\right) \end{array}$$

where *x* is the membrane potential, and *y* and *z* are simplified variables accounting for the sodium and potassium channel activation probabilities. The bifurcation scenario, being a third-order nonlinear dynamical system, with respect to a constant value of *I*, is sufficient to the emergence of chaotic spiking, as shown by the ISI reported in the bifurcation diagram in Figure 11.

The Izhikevich model is based on the following equations:

(7) $$\begin{array}{c} \dot{v}=0.04v^{2}+5v+140-w+I\\ \dot{w}=a\left(bv-w\right) \end{array}$$

with the reset mechanism

(8) $$\begin{array}{c} \mathrm{if}v>30v=c\\ w=w+d \end{array}$$

**Figure 12:**
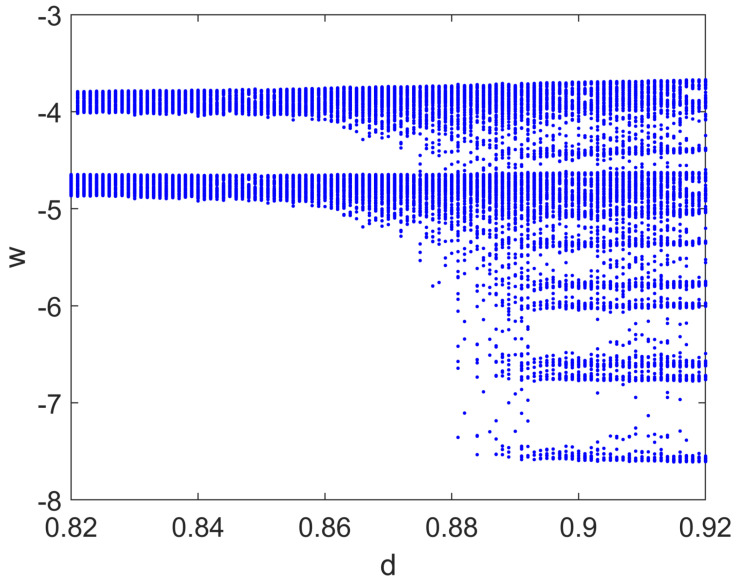
Bifurcation diagram of the Izhikevich model with respect to parameter *d*. Other parameters: a=0.02, b=0.2, c=−55, $I_{m}=10$, $I_{p}=0.1$, ω=0.1 rad/s.

In this case, the bifurcation diagram was obtained by varying the parameter *d* when the input current $I=I_{m}+I_{p}\sin\omega t$, i.e., when a small sinusoidal perturbation was added. The route to irregular and chaotic spiking behaviors is evident in Figure 12.

Finally, the mathematical model of the Morris–Lecar (MS) neuron is reported

(9) $$\begin{array}{c} C_{M}\dot{V}=-g_{L}\left(V-V_{L}\right)-g_{Ca}M_{\infty }\left(V-V_{Ca}\right)-g_{K}N\left(V-V_{K}\right)+I\\ \dot{N}=\frac{N_{\infty }N}{\tau_{N}} \end{array}$$

where

(10) $$\begin{array}{c} M_{\infty }=\frac{1}{2}\left(1+\tanh\frac{V-V_{1}}{V_{2}}\right)\\ N_{\infty }=\frac{1}{2}\left(1+\tanh\frac{V-V_{3}}{V_{4}}\right)\\ \tau_{N}=\frac{1}{\phi \cosh\frac{V-V_{3}}{2V_{4}}} \end{array}$$

**Figure 13:**
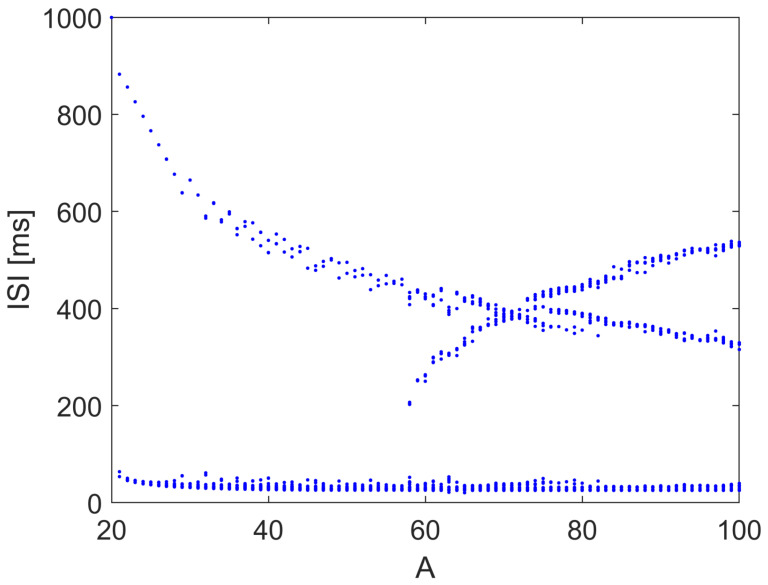
Bifurcation diagram of the Morris–Lecar model with respect to parameter *A*, when I=A(1+sinωt) with ω=0.0063 rad/s. Other parameters: $C_{M}=5$, $g_{K}=8$, $g_{L}=2$, $g_{Ca}=4$, $V_{Ca}=20$, $V_{K}=-80$, $V_{L}=-60$, $V_{1}=-1.2$, $V_{2}=18$, $V_{3}=12$, $V_{4}=17.4$, ϕ=frac115.

In this model, *V* is the membrane potential and *N* accounts for the probability of potassium channel activation. The bifurcation diagram was obtained by varying the amplitude of the sinusoidally varying current *I* as I=A(1+sinωt). Similar to the HH case, as shown in Figure 13, the spiking interval is modulated with small windows of irregular and chaotic spiking times.

## 3. Compact Overview of Recent Literature

This section explains how the papers were selected. We considered an equal distribution of papers that dealt with the considered mathematical models for spiking neurons. Moreover, the selection included very recent papers (at most published in the last ten years).

The research on spiking neurons includes more items. We focused on items more related to nonlinear dynamical systems. The problem of parameter estimation was considered an important point to discuss. Moreover, looking at the intrinsic nonlinear behaviors of neuron models, papers dealing with the important and multidisciplinary topics of bifurcation and chaos were selected. Indeed, the synchronization aspects were considered fundamental, as synchrony in spiking networks leads to information transfer. Attention was devoted to the role of stochastic processes and the positive aspects of noise in spiking neurons. The fundamental issues concerning the implementation of spiking neural models drove the choices of some authors to deal with this aspect. Recent and timely studies on non-integer (fractional) order systems led us to select a series of studies on non-integer order spiking neuron models. Memristors and memristive devices [17] opened up a new area of research in the area of spiking neurons and, therefore, the link between spiking neurons and memristors is highlighted in this review.

In Table 1, the main items and related selected papers are summarized with respect to the specific neuron models considered.

## 4. Focus on the Recent Bibliography on Spiking Systems

**Figure 14:**
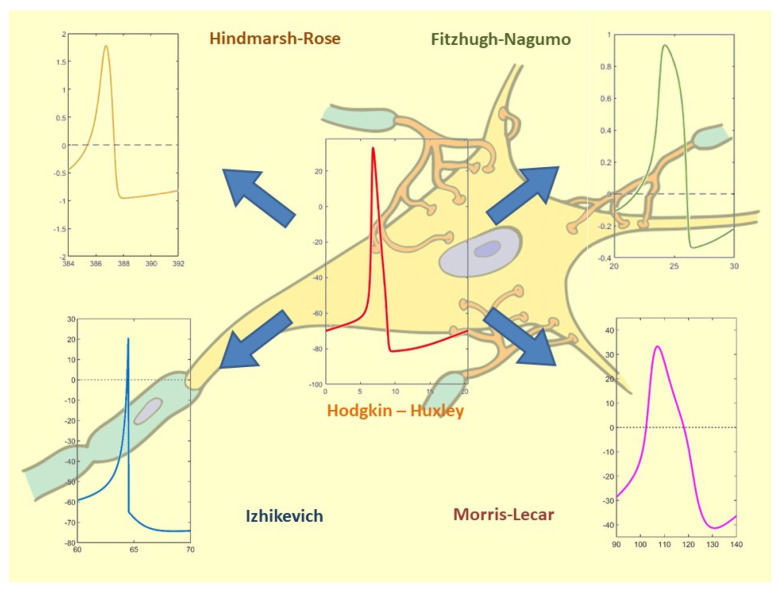
Correlation between spiking neuron models and the selected bibliography. Hodgkin-Huxley: [18,22,23,28,39,46,47,49,50]; Fitzhugh-Nagumo: [19,29,30,31,46,47,51,52]; Hindmarsh-Rose [20,21,24,25,32,33,37]; Izhikevich: [26,34,35,40,41,42,43]; Morris-Lecar: [27,36,38,44,45,48,53,54].

The recent bibliography (organized according to the scheme) is presented in Table 1; the reader can refer to Figure 14 for a complete scenario of the spiking neuron model in each of the selected papers.

### 4.1. Spiking Neuron Parameter Estimation

**Figure 15:**
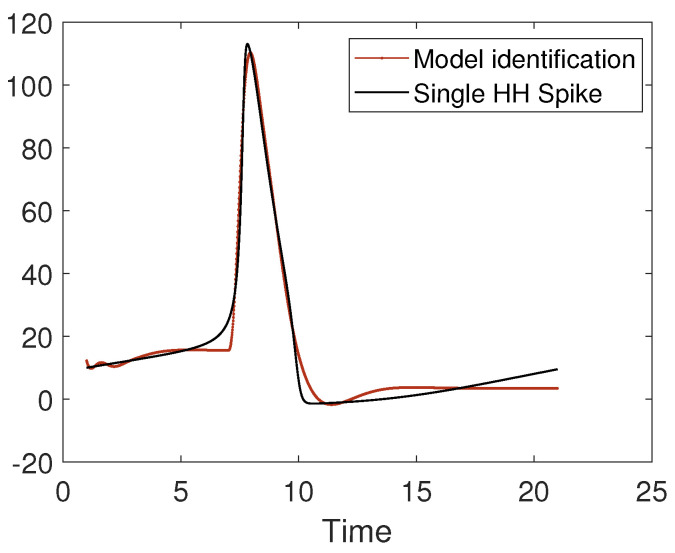
Identification with a nonlinear model of the shape of a single Hodgkin–Huxley spike.

The problem of parameter estimation in a spiking neuron model can be approached with several techniques, including nonlinear autoregressive models [55]. In Figure 15, we present the identification with a nonlinear model of a single Hodgkin–Huxley spike.

The discussion in this subsection refers to recent papers that looked at the problem of parameter identification and the techniques adopted for various spiking neuron models. The various approaches cover different techniques and allow one to successfully determine the model parameters.

In [18], the estimation of HH model parameters is considered. A frequency modulated Möbius model (MMM) is described to define rhythmic patterns in oscillatory spiking systems. Let $x(t_{i})$ with $t_{1}<t_{i}<t_{n}$ be the observed signal. It is modeled in terms of the Möbius phase in the following way:

(11) $$x\left(t_{i}\right)=M+A\cos\phi (t_{i})+e\left(t_{i}\right)$$

with i=1,2,…,n and $M\in R^{+}$, $A\in R^{+}$ and $\phi =\beta +2\arctan\omega \tan\frac{t-\alpha }{2}$, with $\alpha ,\beta \in \left[0,2\pi \right]$ and $\omega \in \left[0,1\right]$, being $e(t_{i})$ the sampling noise.

The signal representation in (11) allows the modulation of the two parameters α and β with the aim of matching the spiking signal. Therefore, by using only the frequency of the spikes and the two parameters α and β, the authors show the modeling sets of HH signals and how to characterize, in a simple way, the neuron membrane potential in several conditions. The paper includes both experimental and simulation results obtained by a specific R program.

Contribution [19] is also related to the model parameter estimation. The neuron model is the FHN model, with four parameters used to characterize its behavior. The neuron is stimulated by using a signal of the form $I=\sum_{i=1}^{N}A_{i}\cos\omega_{n}t+\phi_{n}$. The estimation algorithm is based on the maximum likelihood optimization procedure. Essentially, the authors propose a matching procedure that allows obtaining the four parameters of the described model by making more trials. The optimization algorithm is performed by using Matlab. The success of the study is based on the simplicity gained by using the FHN model. The study is approached as a classic nonlinear identification problem in control applications. The few parameters estimated and the low-order dynamics make the problem easy to be solved. Indeed, the approach was developed in the frequency range from 1000/3 to 10000/3 Hz. The trials give good estimation results. In comparison with the approach used in [18], this is a classic frequency domain parameter identification technique.

The actual interest in neuron parameter estimation was shown in reference [20] where the HR model was considered. The authors assumed that all three measurements for the three state variables of the model were available. Therefore, the proposed approach is based on the minimization of the error between the estimated variables and the measured ones. Of course, the approach is developed in the time domain and the main contribution regards the use of Lyapunov functions in order to guarantee that the norm of the error function converges. The idea involves the use of an observer-based approach to characterize the error system, which, assuming the three variables are measured, can be easily estimated. The use of an adaptive observer allows the neuron parameter estimation.

The results included in reference [21] regard an alternative new approach for the parameter identification of an HR model. Instead of using the set of state-space equations, the authors propose handling the original equations via the Rosenfeld–Gröbner algorithm. The use of differential algebra with a symbolic Maple tool allows for finding a polynomial third-order differential equation. The authors adopted further analytical handling in order to simplify the operator that leads to the input–output relationship containing the estimated parameters. Therefore, the problem is addressed through the identification of a NARMAX model. Indeed, the paper covers another possibility of parameter estimation in the time domain.

The four papers discussed above are quite different in their methods and approaches; moreover, parameter identification techniques of more neuron models were dealt with. Indeed, in [19,20,21], classic identification strategies were successfully adopted; moreover, the use of particular identification techniques for spiking neurons is adopted in [18]. This latter approach appears more original and efficient.

### 4.2. Focus on Bifurcation and Chaos in Spiking Neurons

**Figure 16:**
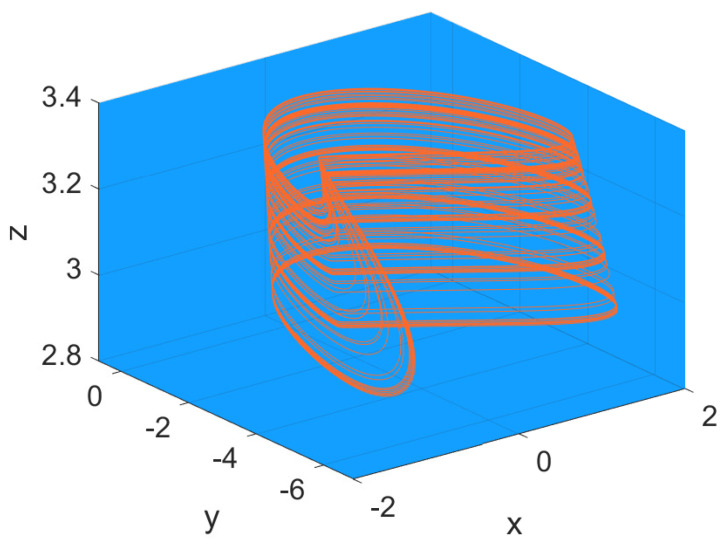
Chaotic attractor in the Hindmarsh–Rose model (6) with parameter values: a=1, b=3, c=1, d=5, r=0.006, s=4, $x_{R}=-\frac{8}{5}$, and I=3.2.

The occurrence of chaotic spiking in neuron models, as in the Hindmarsh–Rose model attractor reported in Figure 16, is a well-established topic which merges neuronal dynamics with nonlinear dynamics and game theory [56]. Even if the topic is described in more of the papers arranged in Table 1, the papers discussed in this subsection show particular results on the specific topic of bifurcation and chaos.

Reference [22] is referred to the HH model and discusses a new phenomenon that is discovered: chaos-delayed decay. It emerges when the first spike of the neuron is delayed by a forcing chaotic signal. The delay depends on the chaos intensity. In the paper, the HH neuron is forced by a chaotic signal generated by the Lorenz system. The phenomenon exists for a set of frequencies from 60 to 150 Hz. The first spike latency and the jitter are evaluated for different frequencies and varying chaos intensities. The study is important for two main reasons: (i) the spike latency is an important mechanism for information processing and coding in neurons, and (ii) a dynamical point of view shows further characteristics of spiking systems in the presence of chaos and how chaotic behavior can control characteristic parameters of spiking signals. The study opens up a new frontier. From a biological point of view, this means evaluating how possible exogenous chaotic signals interfere with neurons. In this sense, pharmacological treatments may generate chaotic activity and, therefore, control neuron mechanisms.

The rich dynamical behavior of spiking HH neurons is further shown in reference [23]. The interference of nonlinear dynamics with stochastic signals has been widely studied in the literature. Moreover, in the case of spiking neurons, this effect induces some peculiar characteristics, such as the stochastic resonance phenomenon and the coherence resonance. The latter is related to the regularization of the spiking response at an optimal noise intensity. In this contribution, the authors remark that, in neurons, we have regularizing effects in more windows (multi-resonance) of noise intensity. In particular, the interspike dynamical behavior was investigated by varying the noise intensities and the regularization effect occurred for two distinct ranges of noise intensity. The study was performed by evaluating the ISI variation. Moreover, a further investigation was performed in order to establish where the double-resonance occurred in the parameter space. The phenomenon arises near a Hopf bifurcation. These interesting remarks led the authors to extend the study to more complex neuron dynamics, taking into account that the original results were derived by using an approximate HH stochastic neuron model. This contribution covers the main features of a spiking signal, as introduced in [3].

In reference [24], the HR neuron model (including a time delay) was considered. The paper was inspired by the experimental results that evaluated the presence of delay in the crayfish stretch receptor organ. The results show the time-delay effect in the coupling of two neurons. The study on synchronization was also performed and discussed.

Spiking neuron models are useful in information technology. Recently, the active use of chaos has achieved a fundamental role in encryption techniques. Reference [25] shows how both the Hopfield model and the spiking HR neuron in chaotic conditions allow the creation of signals that are useful for secure encryption and the possibility of physical implementation by using FPGA techniques. The paper summarizes more results in this area of application.

The essential items related to the complex behaviors of the Izhikevich neuron model are studied in reference [26]. The study was inspired by the research of Hayashi et al. [57] regarding the response of Onchidium giant neurons to the sinusoidal signal. The authors made an accurate analysis of the ISI and its diversity as a function of the amplitude of the sinusoidal input, fixing the frequency. Moreover, a very clear study in the phase plane was performed. This recent contribution explored the complexity of the (very simple) dynamics of the Izhikevich model, showing its emerging bifurcation properties from an experimental perspective.

In reference [27], the authors focused on a short-term memory network for the prediction of the behavior of two coupled ML neurons. Advanced neurons and networks for learning purposes oriented at predicting nonlinear spiking behaviors are challenging. The contribution focuses on the prediction of ML trajectories (starting with little data). This is done by introducing an appropriate recurrent neural network; moreover, small networks can be used to extract local features and correlate them, further reducing the complexity of the prediction network. The study shows that neural networks can fruitfully predict the complex nonlinear behaviors of Morris–Lecar neurons.

### 4.3. Synchronization of Spiking Neurons

**Figure 17:**
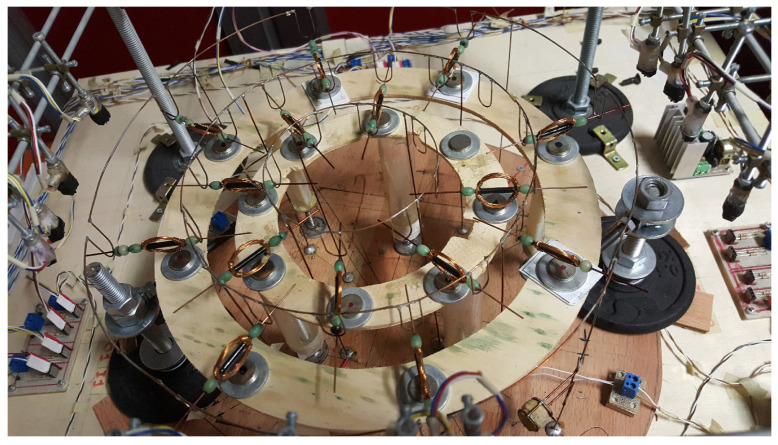
Electromechanical structures of spiking coils.

Synchronization is a general concept that explains how a single unit located in an ensemble can work in phase with others thanks to coupling. As an example, synchronization can be observed in an electromechanical system made of spiking elements, such as that reported in Figure 17, where each element is the coil depicted in Figure 2 [58].

We will now discuss the selected papers that focused on the synchronization of spiking neuron networks. Various techniques were considered; moreover, synchronization approaches are mainly theoretically based on the Lyapunov function of the error and the network topology analysis.

The synchronization of spiking neuron networks is a key area of research. The synchronization of arrays of FHN neurons is presented in reference [29]. The authors presented an easy feedback method to approach synchronization. The strategy is to inject (as control law) the mean value of the signals. Indeed, the control law can be proportional or dynamic. The effects of synchronization and desynchronization were also studied.

In reference [30], a 2D network of FHN neurons with photosensitive excitation was introduced. The master stability function analysis was adopted to discover particular synchronization phenomena. Indeed, chimera states were shown to be possible in the considered network. Spatiotemporal patterns characterized the studied FHN photosensitive network. Their contribution summarizes the concept of collective behavior in complex networks conceived with photosensitive FHN spiking neurons.

Reference [31] considered heterogeneous FHN neurons as the elements of a network with diffusive coupling. The heterogeneous term indicates that the parameters of each neuron are different. The study took into account two FHN neurons and then considered a graph with more nodes. The heterogeneous networks are more difficult to be synchronized with respect to the homogeneous ones. The conditions of possible synchronization were approached by using Lyapunov functions and analytical conditions were ensured by using linear matrix inequalities (LMIs).

Reference [33] mainly covered the role of noise in synchronizing the networks of HR neurons. The paper found the main results of the theory of multi-agent stochastic system synchronization. The authors used the feedback linearization control technique to obtain the control law, making the synchronization error as small as possible and assuring the onset of synchrony. The adopted techniques led to the formulation of the design strategy in terms of LMIs. The numerical results show the suitability of the approach. The HR model is a nonlinear minimum phase system, in terms of the asymptotical stability of its zero-dynamics. This is the key to the success of the presented results.

Reference [34] focused on the synchronization of Izhikevich neurons. The authors considered an external field that could interfere with the neuron’s membrane. They used the Izhikevich model to simulate this particular effect. A further equation was, hence, introduced in the model. The paper presented the bifurcation study for the single neuron and what occurred in the case study of a ring of neurons. The study was performed in various conditions and a variety of patterns were discovered. Moreover, in some cases, chimera states emerged.

In [35], the authors explained how two Izhikevich neurons transmitted information in a unidirectional coupling. Two states, the sub-threshold and the over-threshold, corresponding to the values of the input amplitude, were characterized. The study was performed in the presence of noise and gave a coherent view in terms of biological signals.

Two Morris–Lecar diffusively coupled neurons were considered in reference [36]. The coupling included both weak electrical and chemical signals. The synchronization of these systems was accurately discussed in terms of biophysical behavior. Numerical examples with rich sets of parameters were reported; moreover, even if the study focused on two neurons, it can be extended to complex networks.

### 4.4. Focus on Stochasticity and Noise in Spiking Neurons

The discussion in this subsection is arranged around a strict number of contributions that show how chaotic spiking neurons are supported by stochasticity and noise. This is to emphasize how one theory can reinforce the other.

Reference [28] focused on more items. The first one was the fundamental concept of metabolic action to generate action potentials and maintain neural activity. The second considered the internal noise arising from single ion channels to characterize spiking and critical paths. The third item regarded the coupling of stochastic models with deterministic models. A stochastic Langevin approach (considered the HH model) was adopted to couple the stochastic channel noise and nonlinear dynamics. The path toward synchronization and average energy consumption synthesized the main results of the contribution.

Reference [37] included a new paradigm for the implementation of HR neurons. The authors were inspired by reference [59], where the implementation of a chaotic system was based on stochastic computation. Stochastic computation is an alternative technique used for making the digital computation, as introduced in [60]. Recently, a clear survey on this approach was reported in [61]. The approach in [37] refers to the digital implementation of the HR model; the approximation introduced by the stochastic computing paradigm was considered an imperfection and it could be compensated by adding noise to the device. The study was widely supported by simulations and proved to be an innovative way to synthesize spiking neurons.

In [38], the authors were motivated by the efforts made to approach neural modeling using both deterministic and stochastic models. In particular, the Morris–Lecar model was forced by a non-Gaussian Levy noise current added to the membrane voltage equation. The peculiarity of the Levy noise is that it presents stationarity and independent increments, such as the Brownian motion. The authors aimed to show how the rest condition and the limit cycle condition of the ML dynamics could be switched by the Levy noise process. This is possible due to the similarity characteristic between the Levy noise and the noise related to common neural processes in biological phenomena. The results open up the way for fruitful investigations in in vivo neuron cells.

### 4.5. Focus on Non-Integer Order Spiking Neurons

**Figure 18:**
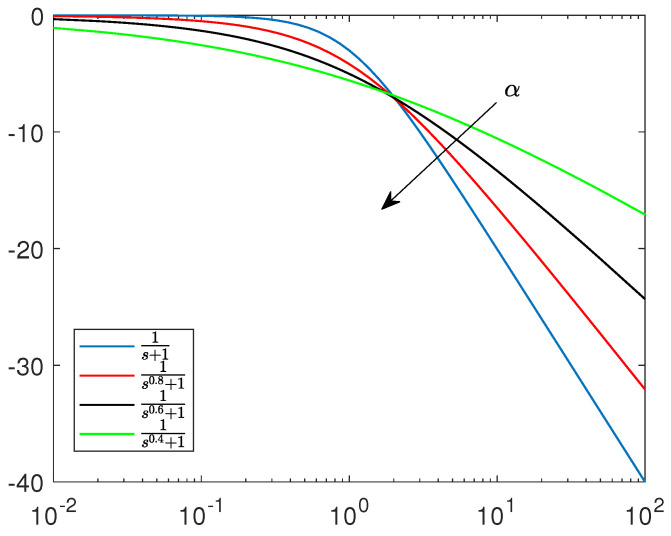
Bode diagram of non-integer order linear systems.

The selected papers discussed here are focused on the timely subject of spiking system dynamics generated by non-integer order dynamical systems. This aspect is, in general, correct, due to the fact that the complexity of spiking neurons cannot be fully covered by integer-order nonlinear models. Non-integer order dynamics actuate the information compression, as emphasized by non-integer order linear system Bode diagrams (reported in Figure 18). This makes the non-integer order spiking neuron models more accurate from a dynamical point of view.

Reference [46] focused on a spatially distributed FHN system with a non-integer order model representation for the time domain and with diffusive second-order terms for the spatial domain. The authors proposed a technique based on the Sumudu transform and the Adomian transform to map the original differential equations. The authors approached the approximation both from theoretical and numerical points of view. This paper makes a link among differential equations in non-integer order systems, advanced approximation strategies, and neuron signal propagation in FHN paths.

The reaction–diffusion (RD) equation is one of the most important equations in science. One of the most popular regards the FHN system, which includes two differential RD equations. Instead of these two equations, a model with only one fractional order differential equation was introduced in [47], where the authors show, with more examples, the appropriateness of the approach.

Reference [32] reports more appealing aspects: (i) the use of HR fractional order systems, (ii) the synchronization, and (iii) the effect of electromagnetic radiation on synchronization. The model was outlined for a pair of neurons and then extended to a ring configuration. The electromagnetic radiation was modeled as a further equation that was assumed of the fractional order *q*. The results include the bifurcation diagrams in terms of the parameter *q*, the study of synchronization in terms of the similarity function, the conditions under which synchronization occurs, and the quantitative analysis of the electromagnetic radiation related to the synchronization.

### 4.6. Focus on Spiking Neurons and Memristors

The memristor was conceived in 1971 by Leon Chua [62] who pointed out that, among the basic components of electrical circuits, a bipole relating to the magnetic flux $\phi_{M}\left(t\right)$ with the amount of charge q(t) was missing. The relationship between the two quantities was expressed by the implicit expression $f(\phi_{M}\left(t\right),q\left(t\right))=0$, with $M=\frac{d\phi_{M}}{dq_{M}}$ being the memristance, measured in Web/C or Ω. Moreover, memristors display typical pinched hysteresis electrical voltage–current characteristics.

Further characteristics of memristors are:

1. The state equations are nonlinear $\dot{x}=f\left(x,q,t\right)$, y=q(x,u,t)u(t);
2. They have bipole memories, called ReRAM;
3. More implementations were recently proposed, yielding different technologies, including titanium dioxide [63], silicon oxide [64], polymeric biomolecular memristor [65], and the self-directed channel memristor [66];
4. They are prone to creating neural synaptic memories, as shown by Crupi et al. [67]

**Figure 19:**
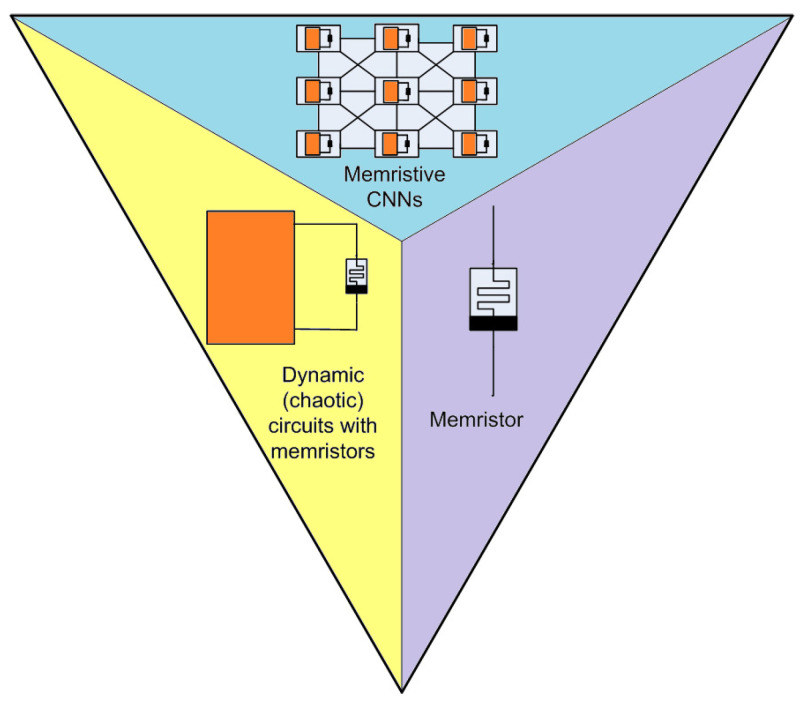
The memristor is the unifying element between nonlinear dynamics and neural networks.

A memristor is considered to be the key element linking neural networks with nonlinear dynamical circuits, as schematically reported in Figure 19.

The previous items are the main ’ingredients’ that have led researchers to consider the memristor as a truly innovative device that can conceive new generations of neuron models. The selected papers discussed herein will focus on this subject, even if the literature on this topic is widely extended.

Reference [49] is the cornerstone contribution; the authors consider the memristor the true innovation device in realizing real HH neurons. A biophysical interpretation of the memristor in terms of the memory device was given, and exact parallelism between the HH model and the Chua Memristor HH model were reported. Moreover, the active membrane models allowed the establishment of the conditions under which the neuron activation could be observed thanks to the memristor behavior. The main concept of the edge of chaos in the Chua Memristor HH model was explained and systems theory-based analytical conditions for spiking were derived.

In reference [50], the authors proposed an electronic circuit with leaky integrate-and-fire behavior mimicking an HH neuron by using two memristor devices, one at the input and one at the output. The memristors were realized using W/WO_3_/PEDOT:PSS/Pt technology. The property of resistive switching of this component was attributed to the migration of protons. The circuit was particularly suited for neuromorphic computing applications.

The authors of [51] referred to the use of memristor devices to realize FHN circuits. The adopted memristor is characterized by a smooth hyperbolic tangent memristance nonlinearity; thus, several chaotic attractors can be generated. The paper included an electronic device that emulated the memristor. A rich discussion on this simple but complex memristor-based neuron was reported.

The state-of-the-art memristor-based neurons and networks were reported on in (the very recent) reference [52], where the authors covered the genesis of memristor studies. The neuron biological characteristics of memristive synapses were presented with accurate biological considerations. Moreover, the role of memristors in realizing artificial neural networks was taken into consideration. The study included a detailed overview of recent memristor technologies.

Besides memristors, other memristive devices were introduced, including existing components. In this context, memcapacitors, i.e., capacitors with nonlinear characteristics, are used in innovative spiking neuron models. In [54], memcapacitors were adopted to implement spiking neurons, allowing for a dynamically evolving time constant to obtain a long-term memory device.

### 4.7. Focus on the Implementation of Spiking Neurons

**Figure 20:**
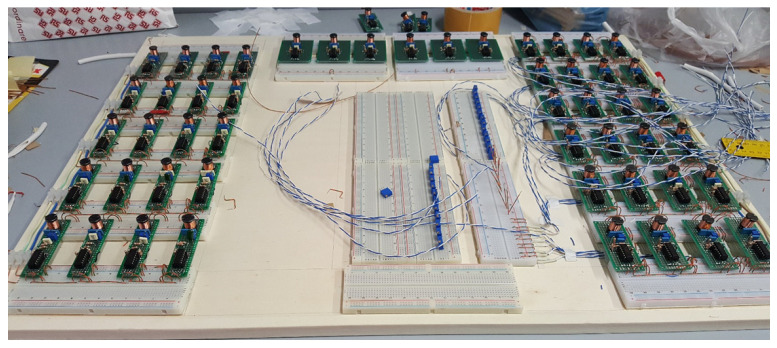
Analog implementation of a large-scale network of spiking systems based on integrated devices.

Implementing large-scale networks of spiking neurons is a fundamental topic in view of practical real-time applications. Solutions based on analog devices, such as the network reported in Figure 20, allow for fast observations of the spiking dynamics and play fundamental roles in educational tasks. The literature, therefore, is very rich in this particular area. The selected papers focused on the considered five models, taking into account the various technologies that were used to implement them.

Even if there has recently been a lot of activity in realizing HH neurons, due to the advances in CMOS technology [68], reference [39] is quite appealing. The authors proposed an electronic circuit that was very easy to be reproduced, which mimicked the dynamics of an HH neuron. The circuit can be used for research aims and education. Moreover, the structure of the device is suitable for neuronal network implementations.

Image encryption by using chaotic signals is a timely topic. In reference [25], the authors propose an approach based on Hopfield and HR neurons. After discussing the bifurcation scenarios of the system, the FPGA technology was adopted for implementation. The paper presented a further application of spiking neurons for advanced information technology applications. This means that real neuro-inspired efficient systems are reliable systems that could be easily realized with low-cost implementations.

In reference [40], the authors focus on the ultra-compact neuron (UCN) realization, providing an accurate discussion of the various technologies that can be useful for spiking neuron implementation. An UCN consists of two transistors and one silicon-controlled rectifier (SCR). The authors remarked that this special spiking neuron configuration mimics more biological neuron behaviors. Moreover, the simplicity of the electronic scheme and the possibility of its implementation in MOSFET technology opens up the way for the realization of networks made of a relevant number of UCNs.

Reference [41] focused on the implementation of an Izhikevich neuron. The authors focused on a modification of the classic Izhikevich mathematical model in order to further reduce the hardware digital implementation. The simplicity of the silicon requirements (with respect to the previous realization) allows for the implementation a higher number of spiking neurons that work in parallel. The authors compared it with the original Izhikevich implementation in order to validate their simplification.

In [42], the authors focus on a complete dynamical model of spiking neurons to outline the basic elements of a reliable FPGA implementation of an Izhikevich neuron. The study highlights the implementation costs that—thanks to the FPGA techniques—can be reduced.

Reference [43] concerns the Izhikevich model implementation. The authors presented the detailed state-of-the-art and a new approach to realizing Izhikevich neurons. They proposed a lookup table approach that was easy to implement and very fast. The realization of the proposed architecture allows for the reproduction of twenty Izhikevich neurons in low-cost FPGA resources. The evaluation of the implementation confirms the high-speed performance.

The Morris–Lecar neuron model is proposed in [44]. The authors proposed a new device to generate the exponential terms and the FPGA implementation. The results are very similar to those simulated with Matlab.

According to the authors of [45], the Morris–Lecar spiking neuron can be easily realized by using analog circuits. An innovative design formulation allows using few elements and the use of the advanced MOS 45 nm technology. The design appears robust with respect to the technology process variability.

## 5. Concluding Remarks

The motivation for this review was to provide the reader with a recent scenario of the fascinating topic of spiking neurons. Moreover, we were motivated by the recent papers related to in silico neuron implementation, with low-cost platforms, such as references [69,70], which are related to spiking neuron networks. Moreover, we were also interested in the implementation of high-order artificial spiking systems, so-called hyperneurons [71].

There was impressive research made in recent years related to the topic. Regarding the theoretical aspects, the following topics are still considered of further interest: the interaction between spiking membrane dynamics and random processes and the active role of noise in spiking regularization and describing impressive dynamical behaviors.

The analytical aspects related to the synchronization conditions reveal the important role of the Lyapunov theory and the LMI techniques. Indeed, more activities on synchronization involve the network theory. The practical implementation of the various spiking neurons involves more advanced electronic hardware techniques. The implementation research involves both analog and digital components. This means that both simple analog discrete component devices and VLSI analog technology were used. Moreover, techniques both on VLSI digital design and FPGA platforms are proposed for digital implementations. To encourage the reader, it is important to understand that more spiking neurons and their applications only require low-cost laboratory equipment.

Memristor systems will open up the way to further robust spiking neuron implementations. The spiking neuron subject includes more topics from biophysics and physics. The theme is quite open and includes a wide range of disciplines from chemistry to mathematics; in the area of mathematics, it includes everything from applied mathematics to differential geometry.

Spiking neurons involve new techniques and theories that can be seen as challenging topics in the next century in neuroscience–computing science. Spiking systems are robust, in the sense that they are noise-insensitive, even if this noise can be useful in regularizing their behavior [23]. Moreover, in a general sense, spiking systems are often characterized by the emergence of self-organization, such as electromechanical spiking systems [58]. The positive role of noise in neural models and neural architectures is an intriguing topic that deserves deeper investigations.

Spiking systems can be used for implementing pattern generators for bioinspired robotic systems [72]. Indeed, the use of spiking signals for control purposes is a topic that is gaining renewed interest [73]. Its roots are in neuromorphic engineering [74] and spiking artificial neural networks [75]. These structures are designed with the aim of providing high efficiency in solving complex problems, including technological problems with low-power consumption. A future trend in spiking system research involves the adoption of reliable event-based technologies [76], due to the fact that spiking signals are analog signals with intrinsic digital nature (due to the enumerability of spikes). The possibility of using spiking models in the context of brain–computer interfaces for advanced control purposes appears to be worthy of investigation. Finally, in the context of realizing high-dimensional and robust networks of spiking neurons, ‘hypersystems’ [71] are opening up the doors for further investigations into emerging collective behaviors in large ensembles of spiking neurons.

## Figures and Tables

**Table 1:** Correlation between topics and spiking neuron models in the selected bibliographies.

	HH	FHN	HR	Izhikevich	ML
Parameter Estimation	[18]	[19]	[20,21]		
Bifurcation and Chaos	[22,23]		[24,25]	[26]	[27]
Synchronization	[28]	[29,30,31]	[32,33]	[34,35]	[36]
Stochasticity and Noise	[23,28]		[33,37]		[38]
Implementations	[39]		[25,37]	[40,41,42,43]	[44,45]
Non-integer order		[46,47]	[32]		[48]
Memristors	[49,50]	[51,52]			[53,54]

## Data Availability

Data are not available.
